# Which lymph node dissection template is optimal for radical cystectomy? A systematic review and Bayesian network meta-analysis

**DOI:** 10.3389/fonc.2022.986150

**Published:** 2022-11-25

**Authors:** Wenqiang Qi, Minglei Zhong, Ning Jiang, Yongheng Zhou, Guangda Lv, Rongyang Li, Benkang Shi, Shouzhen Chen

**Affiliations:** ^1^ Department of Urology, Qilu Hospital of Shandong University, Jinan, China; ^2^ Department of Epidemiology, School of Public Health, Cheeloo College of Medicine, Shandong University, Jinan, China; ^3^ Department of Thoracic Surgery, Qilu Hospital of Shandong University, Jinan, China

**Keywords:** bladder cancer, pelvic lymph node dissection, prognosis, complications, Bayesian analysis

## Abstract

**Objective:**

This study aims to determine the optimal pelvic lymph node dissection (PLND) template for radical cystectomy (RC).

**Methods:**

A systematic search was conducted using the PubMed, Embase and Cochrane Library database in December 2021. Articles comparing recurrence-free survival (RFS), disease-specific survival (DSS), overall survival (OS), and postoperative complications among patients undergoing limited PLND (lPLND), standard PLND (sPLND), extended PLND (ePLND), or super-extended PLND (sePLND) were included. A Bayesian approach was used for network meta-analysis.

**Results:**

We included 18 studies in this systematic review, and 17 studies met our criteria for network meta-analysis. We performed meta-analyses and network meta-analyses to investigate the associations between four PLND templates and the RFS, DSS, OS, or postoperative complications. We found that the ePLND group and the sePLND group were associated with better RFS than the sPLND group (Hazard Ratio [HR]: 0.65, 95% Credible Interval [CrI]: 0.56 to 0.78) (HR: 0.67, 95% CrI: 0.56 to 0.83) and the lPLND group (HR: 0.67, 95% CrI: 0.50 to 0.91) (HR: 0.70, 95% CrI: 0.49 to 0.99). For RFS, Analysis of the treatment ranking revealed that ePLND had the highest probabilities to be the best template. There was no significant difference between the four templates in DSS, however, analysis of the treatment ranking indicated that sePLND had the highest probabilities to be the best template. And We found that the sePLND group and the ePLND group were associated with better OS than lPLND (HR: 0.58, 95% CrI: 0.36 to 0.95) (HR: 0.63, 95% CrI: 0.41 to 0.94). For OS, analysis of the treatment ranking revealed that sePLND had the highest probabilities to be the best template. The results of meta-analyses and network meta-analyses showed that postoperative complications rates did not differ significantly between any two templates.

**Conclusion:**

Patients undergoing sePLND and ePLND had better RFS but not better DSS or OS than those undergoing lPLND or sPLND templates, however, RFS did not differ between patients undergoing sePLND or ePLND. Considering that sePLND involves longer operation time, higher risk, and greater degree of difficulty than ePLND, and performing sePLND may not result in better prognosis, so it seems that there is no need for seLPND. We think that ePLND might be the optimal PLND template for RC.

**Systematic Review Registration:**

https://www.crd.york.ac.uk/prospero/, identifier CRD42022318475.

## 1 Introduction

Bladder cancer (BCa) is one of the most common malignant tumors of the urinary system. It is ranked as the 7th most malignant tumor in males and the 10th in both genders (1). About 25% of patients with BCa present with muscle-invasive bladder cancer (MIBC), which is associated with lower survival rates than patients with non-muscle-invasive bladder cancer (NMIBC) (2, 3).

Radical cystectomy (RC) is an important part of the standard treatment for MIBC around the world (4, 5). RC refers to the removal of the bladder together with wide excision of the soft tissue around the bladder, including the pelvic lymph nodes (6). A reasonable pelvic lymph node dissection (PLND) template is crucial to assess the status of pelvic lymph nodes and predicting the prognosis of patients (6).

However, due to the inconsistent and biased nature of current studies, the optimal extent of PLND remains controversial. For a long time, there was no clear definition of the boundaries of different PLND templates. Based on the European Association of Urology (EAU) guidelines (2021) and other studies, we clarified the following definitions for different PLND templates. Only obturator and internal iliac nodes were resected in limited PLND (lPLND) template (7). A standard PLND (sPLND) template is removal of internal iliac, presacral, obturator fossa, and external iliac lymph nodes (8). All lymph nodes in the region of the aortic bifurcation, presacral and common iliac vessels medial to the crossing ureters are resected in an extended PLND (ePLND) template. The lateral borders are the genitofemoral nerves, caudally the circumflex iliac vein, the lacunar ligament, and the lymph nodes of Cloquet, as well as the area described for sPLND (8–12). A super-extended PLND (ePLND) template extends to the level of the inferior mesenteric artery (13, 14).

A wider extent of lymph node dissection might be associated with a better survival outcome (15). However, excessive lymph node dissection is associated with longer operation time, higher risk, and greater degree of difficulty, which can be harmful to patients (14). Therefore, the aim of our study was to identify an optimal PLND template and to avoid excessive lymph node dissection.

## 2 Methods

The study was conducted according to the Preferred Reporting Items for Systematic Reviews and Meta-analyses (PRISMA) extension statement for network meta-analysis (16), and it has been registered at the International Prospective Register of Systematic Reviews (PROSPERO; https://www.crd.york.ac.uk/prospero/) as registration number CRD42022318475.

### 2.1 Search strategies

A systematic search was conducted using the PubMed, Embase, Cochrane library database (up to December, 2021). Search strategies were designed to include patients with BCa who underwent RC and different extents of PLND. Terms and keywords such as “radical cystectomy” “RC” “lymphadenectomy” “lymph node dissection” and “LND” were used to conduct the search. The detailed search strategies are shown in **Supplementary Material S1**. Two authors (Wenqiang Qi and Minglei Zhong) independently reviewed and cross-checked all articles. In addition, electronic searches were supplemented by manual searches of the reference lists of relevant articles.

### 2.2 Selection criteria

Studies published in English were included if they met the following criteria: (1) Patients with BCa. (2) The included manuscripts should include comparisons of at least two templates of lPLND, sPLND, ePLND or sePLND in their analyses. (3) Full-text articles and data on survival outcomes and complications were available.

The exclusion criteria were: (1) Case reports, reviews, conference abstracts, and other ineligible article types. (2) No survival outcome or complications rate. (3) Written in languages other than English.

### 2.3 Data extraction

Two reviewers (Wenqiang Qi and Minglei Zhong) independently extracted the following information from the included studies: author’s name, publication year, study design, study population, PLND templates, age, pathological stage, lymph nodes status, neoadjuvant or adjuvant therapy, follow-up time, recurrence-free survival (RFS), disease-specific survival (DSS), overall-survival (OS) and complications rates. All disagreements regarding data extraction were resolved by discussions with the third reviewer (Yongheng Zhou).

### 2.4 Outcome measures

The main outcome is RFS. The secondary results are DSS, OS and complications rates.

### 2.5 Quality assessment

We evaluated the quality of each cohort study using the Newcastle-Ottawa Quality Assessment Scale (NOS), and studies with scores equal to or higher than 6 could be included in our meta-analyses and network meta-analyses (17). The methodological quality of each randomized controlled trial (RCT) was evaluated using the Cochrane collaboration’s tool (version 5.3, The Nordic Cochrane Centre, The Cochrane Collaboration, USA) (18). The tool includes seven aspects to assess deviation risk.

Two investigators (Wenqiang Qi and Minglei Zhong) conducted quality assessment independently. Disagreements were solved through discussions with the third investigator (Yongheng Zhou).

### 2.6 Statistical analyses

The RFS, DSS and OS results are represented by Hazard Ratios (HRs) and their 95% Credible Intervals (CrIs). For studies only with survival curves, we collected results from the curves by Engauge Digitizer V4.1 (Markmitch, Goteborg, Sweden) (19). The random effects model was used for the network meta-analysis of RFS, DSS and OS results. The model uses group-level information and models (log) HRs to preserve randomization and accounts for within-trial correlation of multigroup trials (20). It was implemented within a Bayesian framework using OpenBUGS (version 3.2.3). The code we used in OpenBUGS is shown in **Supplementary material S2**. The median of the result can be used as the point estimation of the treatment effect, and after ensuring the distributions are approximately normally distributed, a 95% CrI is derived from the 2.5th and 97.5th percentiles (20). In addition, we derived the probabilities of the four PLND rankings for survival outcomes from the calculation results. The inconsistency is the difference between direct and indirect evidence, for ease of interpretation, we expressed inconsistency as the percentage difference in HRs between direct and indirect comparisons. The larger the absolute value, the larger the inconsistency. Values of ± 25%, ± 50%, and ± 100% represent low, medium, and substantial inconsistency, respectively (21). The complications rates of each group are represented by Odds Ratios (ORs) and their 95% CrIs. We compared the complications rates of different templates using ADDIS (Aggregate Data Drug Information System) software V1.16.8. Network meta-analysis uses a Bayesian approach and allows comparisons among all PLND templates. We adopted random effects model to perform the most appropriate and conservative analysis. The node splitting analysis was used to study the inconsistency between direct and indirect comparisons, p values > 0.05 reveal that there is no inconsistency in the network. We also derived the probabilities of the four PLND rankings for complications rates from the calculation results.

We calculated the HRs with 95% Confidence Intervals (CIs) to summarize the effects of the survival outcomes in direct comparison, and the ORs with 95% CIs were calculated to summarize the complications rates. A 2-sided p-value of less than 0.05 was defined as statistical significance. In our research, the random effects model was used to estimate the size of the collective effect to reduce possible deviations. All direct comparisons were performed using Review Manager software (Revman version 5.3, Nordic Cochrane Center, Cochrane Collaboration, 2014).

## 3 Results

### 3.1 Literature search

**Figure 1:**
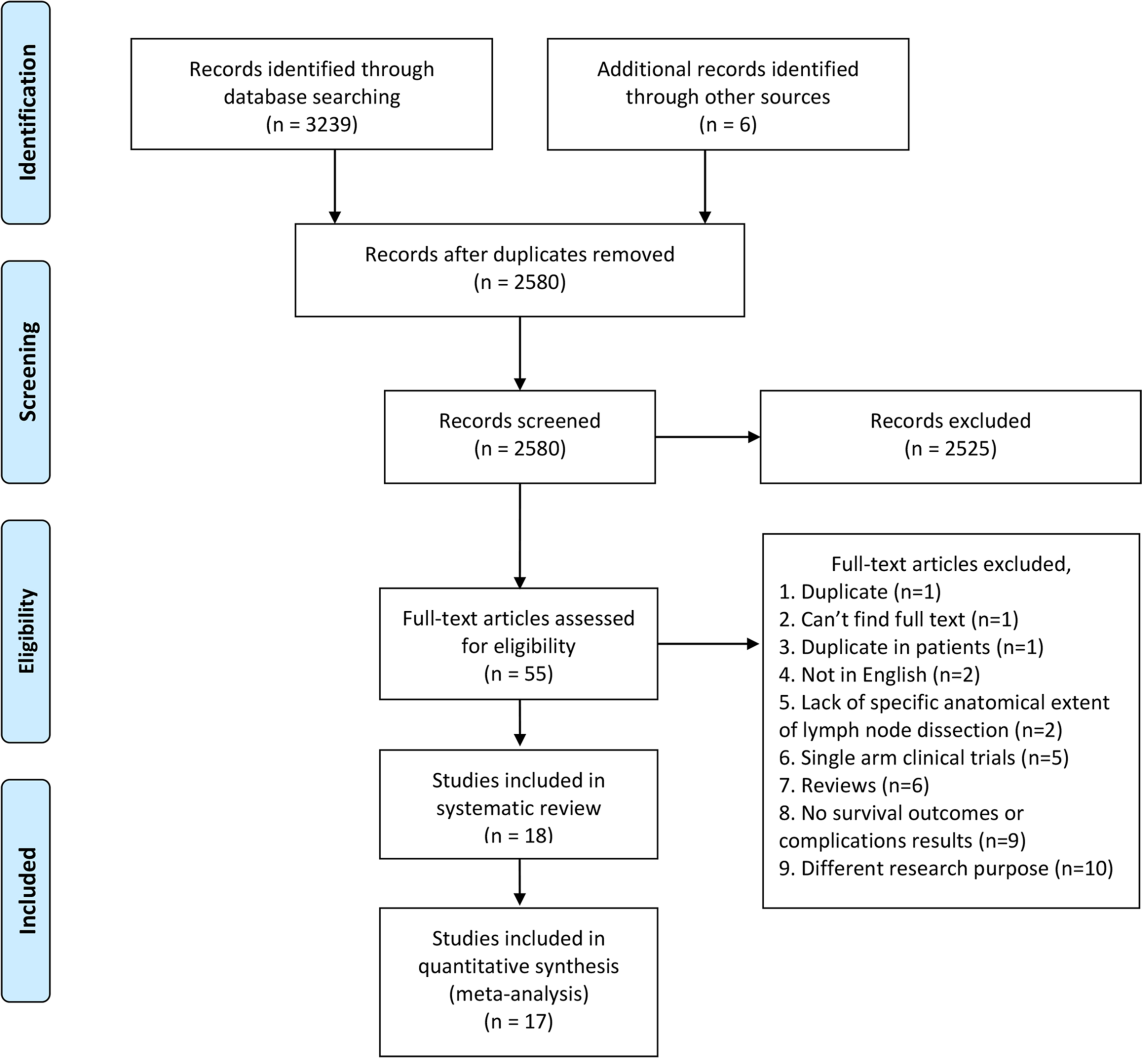
PRISMA flow diagram of literature retrieval. PRISMA, Preferred Reporting Items for Systematic Reviews and Meta-Analyses.

The flowchart of the literature search is shown in **Figure 1**. After the initial search, we identified 3245 relevant articles. We excluded 665 articles because of duplication. After screening titles and abstracts, 2525 articles were excluded because of unrelated outcomes. Fifty-five full-text articles were assessed, and 18 (8–10, 12–14, 22–33) of them were included in our study, 17 (8–10, 12–14, 22–31, 33) eligible studies were included in quantitative analysis. One study (32) had a NOS score less than 6, therefore we excluded it from quantitative analysis to avoid biasing our conclusions.

### 3.2 Information of included studies and patients

The detailed patients’ characteristics are summarized in **Table 1**. A total of 6503 patients were enrolled in our quantitative analysis, including 660 patients in the lPLND group, 2165 patients in the sPLND group, 2206 patients in the ePLND group, and 1472 patients in the sePLND group.

**Table 1:** Baseline characteristics of included studies.

Authors	Year	Study design	Type of PLND (C/I or C/I^1^/I^2^)				Participants (C/I or C/I^1^/I^2^)				Age (C/I or C/I^1^/I^2^)				Pathological stage (C/I or C/I^1^/I^2^)				LN status (C/I or C/I^1^/I^2^)				Number of LNs yield (C/I or C/I^1^/I^2^)				Neoadjuvant or adjuvant therapy		Follow up months
Poulsen et al.	1998	Cohort study	sPLND		ePLND		68		126		63.2(30.2-74.21		61.8(27.2-81.9)		T0-a-is: 8/T1: 17/T2: 9/T3: 31/T4: 2		T0-a-is: 26/T1: 16/T2: 13/T3: 62/T4: 7		N0: 53/N+: 15		N0: 91/N+: 35		14 (5–30)		25 (9–67)			Palliative chemotherapy was used in recurrence cases	61.7/23.5
Brössner et al.	2004	Cohort study	lPLND		sePLND		46		46		68.2(51-83)		66.3(46-81)		T1:6/T2-3a:18/T3b:22		T1:4/T2-3a:24/T3b:18		N0:36 N+:10		N0:28 N+:18		NR		NR			NR	NR
Dhar et al.	2008	Cohort study	lPLND		ePLND		336		322		61.6 (32-84)		66.9 (35-89)		T2: 200/T3: 136		T2: 150/T3: 172		N0: 292/N+: 44		N0: 239/N+: 83		12(2–31)		22(10–43)			None	51/36
Holmer et al.	2009	Cohort study	lPLND		ePLND		69		101		68 (39-79)		66 (46-83)		T0-a-is: 20/T1: 7/T2: 19/T3: 16/T4a: 7		T0-a-is: 21/T1: 10/T2: 22/T3: 40/T4a: 8		N0: 57/N+: 12		N0: 63/N+: 38		8 (1–36)		37 (8–71)			ACT was used in 25 patients (~15%)	94/38
Hugen et al.	2010	Cohort study	sPLND		sePLND		206		54		NR		NR		Tis:166/not Tis:87/NR:7				N0-Nx:206		N0-Nx:54		9(0–74)		46(9–112)			NR	60
Abol-Enein et al.	2011	Cohort study	sPLND		sePLND		200		200		50.5 (12.0)		55.0 (12.0)		Tis, T1: 29/T2: 32/T3-4: 139		Tis, T1: 23/T2: 47/T3-4: 129		N0: 152/N+: 48		N0: 152/N+: 48		16 (8.0)		49.0 (18.75)			None	50.2
Dharaskar et al.	2011	Cohort study	sPLND		ePLND		23		27		NR		NR		T0: 2/Ta-1: 4/T2: 12/T3: 4/T4: 1		T0: 2/Ta-1: 5/T2: 13/T3: 6/T4: 1		N0: 17/N+: 6		N0: 20/N+: 7		9 (3–28)		16 (1–25)			MVAC/GC chemotherapy in LN+ and/or T3/T4 patients	14 (0–43)/6 (0–37)
Zehnder et al.	2011	Cohort study	ePLND		sePLND		405		554		67 (36–89)		67 (31–91)		T2: 169/T3: 236		T2: 253/T3: 301		N0: 291/N+: 114		N0: 359/N+: 195		22 (10–60)		38 (10–179)			ACT was used in 241 patients (~25%)	9.9y/10.9y
Zhu et al.	2012	Cohort study	lPLND		sPLND		112		134		<60y:99/60-69y:86/≥70y:61				T ≤ 2: 127/T3: 91/T4: 28				NR		NR		NR		NR			None	47 ± 31
Simone et al.	2013	Cohort study	sPLND		ePLND		584		349		66.9 ± 9.2		65.4 ± 8.7		T0-a-is-1: 140/T2: 131/T3: 235/T4: 78		T0-a-is-1: 94/T2: 98/T3: 108/T4: 49		N0: 397/N+: 187		N0: 242/N+: 107		16.6 ( ± 11.8)		32.7( ± 14.9)			ACT (~11%) and RT (~1%) in some patients	96
Mata et al.	2015	Cohort study	sPLND		ePLND		224		205		62 (IQR 54-70).				NR		NR		NR		NR		14 (10-19)		36 (21-56)			Adjuvant MVAC in some patients	NR
Abdi et al.	2016	Cohort study	sPLND		ePLND		105		105		68.7 (± 10.3)		68.4 (± 9.0)		T0-a-is-1: 47/T2: 8/T3: 32/T4: 18		T0-is-a-1: 53/T2: 8/T3: 28/T4: 16		N0: 91/N+: 14		N0: 87/N+: 18		9 (6-12)		21 (13-29)			NACT in 50 patients (23.8%)	18/19
Møller et al.	2016	Cohort study	ePLND		sePLND		316		262		69 (30–91)		64 (34–85)		Ta-is-x: 16/T1: 65/T2: 121/T3: 86/T4a:27		Ta-is: 9/T1: 48/T2: 99/T3: 79/T4a:27		NR		NR		NR		NR			None	38/93
Gschwend et al.	2019	RCT	sPLND		sePLND		203		198		68 (61-73)		67 (59-74)		T1: 24/T2: 81/T3: 68/T4: 30		T1: 31/T2: 88/T3: 63/T4: 16		N0: 147/N+: 56		N0: 152/N+: 44/Nx:2		19 (12-26)		31 (22-47)			ACT in 58 patients (14.5%)	43
Brunocilla et al.	2013	Cohort study	lPLND		sPLND		97		94		68.3 ± 8.3				NR				N0:71/N+:26		N0:66/N+:28		8.3 ± 3.8		18.1 ± 4.1			Palliative chemotherapy and/or RT in recurrence cases	59.2 ± 44.3 (1-171)
Choi et al.	2019	Cohort study	sPLND	ePLND		sePLND	124	216		108	63.4 ± 10.4	63.9 ± 9.5		63.6 ± 9.9	≤T2:65/≥T 3:59	≤T2:114/≥T3:102		≤T2:46/≥T3:62	N0:115/N+:9	N0:189/N+:27		N0:91/N+:17	14.5 ± 7.6	25.3 ± 11.4		42.5 ± 15.7		None	41.6
D’Andrea et al.	2020	Cohort study	sPLND	ePLND		sePLND	200	34		50	67(61-74)	65(62-73.7)		68(57.5-75.7)	Tis-a-1:47 /T0:1/T2:57/T3:62/T4:33	Tis-a-1:9 /T0:1/T2:8/T3:10/T4:6		Tis-a-1:14 /T0:5/T2:11/T3:17/T4:3	N0:143/N+:57	N0:25/N+:9		N0:33/N+:17	13 (7-22)	31 (13-37)		39 (25-52)		NACT in 25 patients (8.8%)	NR

PLND, Pelvic Lymph Node Dissection; C, the Control group; I^1^, the Intervention group 1; I^2^, the Intervention group 2; LNs, Lymph Nodes; sPLND, standard PLND; ePLND, extended PLND; sePLND, super-extended PLND; ACT, Adjuvant Chemotherapy; NR, Not Reported; RT, Radiotherapy; IQR, Interquartile Rang; NACT, Neoadjuvant Chemotherapy; RCT, Randomized Controlled Trial.

### 3.3 Results of the quality assessment

The quality assessment results of the cohort studies are shown in **Supplementary Material S3**. Except for one study (32), the scores of other studies were equal to or higher than 6. The quality assessment results of the RCT (29) are shown in **Supplementary Material S4**. Apart from performance bias and detection bias, the risk of other bias was not high.

### 3.4 Results of direct comparison

#### 3.4.1 RFS

**Figure 2:**
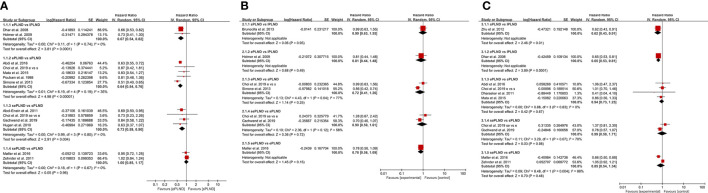
**(A)** Forest plot for direct comparisons of RFS in patients with different PLND templates. **(B)** Forest plot for direct comparisons of DSS in patients with different PLND templates. **(C)** Forest plot for direct comparisons of OS in patients with different PLND templates. RFS, Recurrence free survival. PLND, Pelvic lymph node dissection. DSS, Disease specific survival. OS, Overall survival. CI, confidence interval. lPLND, limited pelvic lymph node dissection. sPLND, standard pelvic lymph node dissection. ePLND, extended pelvic lymph node dissection. sePLND, super-extended pelvic lymph node dissection.

Two studies (9, 10) assessed the difference in RFS between the ePLND group and the lPLND group, and the pooled result showed that ePLND was associated with better RFS (HR: 0.67, 95% CI: 0.54 to 0.82, p=0.0001). Five studies (8, 12, 22, 26, 31) assessed the difference in RFS between the ePLND group and the sPLND group, and the pooled result showed ePLND was associated with better RFS (HR:0.64, 95% CI: 0.54 to 0.76, p<0.00001). Four studies (23, 26, 29, 30) reported the difference in RFS between the sePLND group and the sPLND group, and the pooled result showed sePLND was associated with better RFS (HR: 0.73, 95% CI: 0.59 to 0.90, p=0.004). Two studies (13, 14) reported the difference in RFS between the sePLND group and the ePLND group, and the pooled result showed that there was no significant difference in RFS between the two groups (HR: 1.00, 95% CI: 0.85 to 1.17, p=0.96). (**Figure 2A**).

#### 3.4.2 DSS

Two studies (8, 26) reported the difference in DSS between the ePLND group and the sPLND group, and there was no significant difference in DSS between the two groups (HR: 0.72, 95% CI: 0.41 to 1.26, p=0.25). Two studies (26, 29) reported the difference in DSS between the sePLND group and the sPLND group, and the pooled result showed that there was no significant difference in DSS between the two groups (HR: 0.90, 95% CI: 0.50 to 1.61, p=0.72). Only one study assessed the difference in DSS between the sPLND group and the lPLND group (25), and the situation was similar between the ePLND group and the lPLND group (10), and between the sePLND group and the ePLND group (13). The pooled results indicated that there was no evidence to show that one treatment was associated with better DSS than the other. (**Figure 2B**)

#### 3.4.3 OS

Four studies (22, 26, 28, 31) reported the difference in OS between the ePLND group and the sPLND group, and there was no significant difference in OS between the two groups (HR: 0.94, 95% CI: 0.73 to 1.23, P=0.67). Two studies (26, 29) assessed the difference between the sePLND group and the sPLND group, and the pooled result showed that there was no significant difference in OS between the two groups (HR: 0.99, 95% CI: 0.58 to 1.71, p=0.98). Two studies (13, 14) assessed the difference between the sePLND group and the ePLND group, and the pooled result showed there was no significant difference in OS between the two groups (HR: 0.85, 95% CI: 0.54 to 1.34, p=0.48). Only one study (33) assessed the difference in OS between the sPLND group and the lPLND group, and the results of this study showed that sPLND was associated with better OS. And only one study (9) assessed the difference in OS between the ePLND group and the lPLND group, the results showed that ePLND was associated with better OS. (**Figure 2C**)

#### 3.4.4 Complications

**Figure 3:**
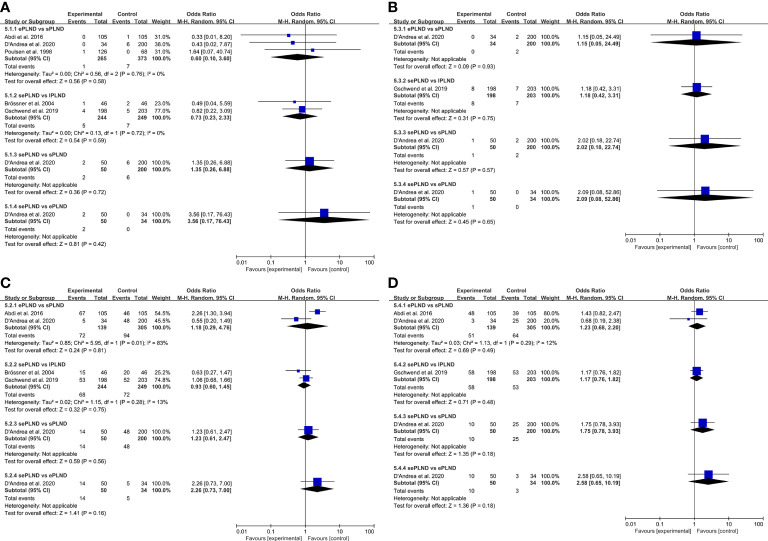
**(A)** Forest plot for direct comparisons of early Clavien-Dindo grade 5 complications rates in patients with different PLND templates. **(B)** Forest plot for direct comparisons of late Clavien-Dindo grade 5 complications rates in patients with different PLND templates. **(C)** Forest plot for direct comparisons of early Clavien-Dindo grade 3-4 complications rates in patients with different PLND templates. **(D)** Forest plot for direct comparisons of late Clavien-Dindo grade 3-4 complications rates in patients with different PLND templates. PLND, Pelvic lymph node dissection. CI, confidence interval. lPLND, limited pelvic lymph node dissection. sPLND, standard pelvic lymph node dissection. ePLND, extended pelvic lymph node dissection. sePLND, super-extended pelvic lymph node dissection.

We graded complications according to the modified Clavien-Dindo classification (CDC), and we only focused on the major complications (CDC grade ≥3), of which grade 5 represents death of a patient (34). Five studies reported major complication rates among different groups, and there was no significant difference in early or late major complications between any two groups (**Figure 3**).

### 3.5 Results of Bayesian analyses

**Figure 4:**
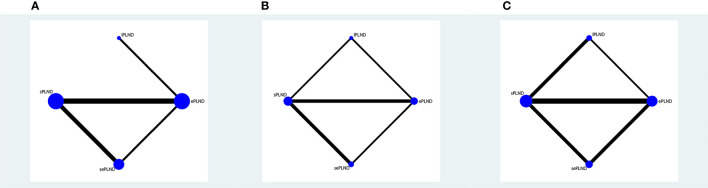
**(A)** Network plot showing the association of different PLND templates with the RFS in patients undergoing radical cystectomy. **(B)** Network plot showing the association of different PLND templates with the DSS in patients undergoing radical cystectomy. **(C)** Network plot showing the association of different PLND templates with the OS in patients undergoing radical cystectomy. PLND, Pelvic lymph node dissection. RFS, Recurrence free survival. PLND, Pelvic lymph node dissection. DSS, Disease specific survival. OS, Overall survival. lPLND, limited pelvic lymph node dissection. sPLND, standard pelvic lymph node dissection. ePLND, extended pelvic lymph node dissection. sePLND, super-extended pelvic lymph node dissection. Note: Lymph node dissection templates are represented by nodes and direct comparison trials between different templates are linked with a line. The area of the dot represents the sample size of each template and the width of the line corresponds the number of direct comparison trials.

**Figure 5:**
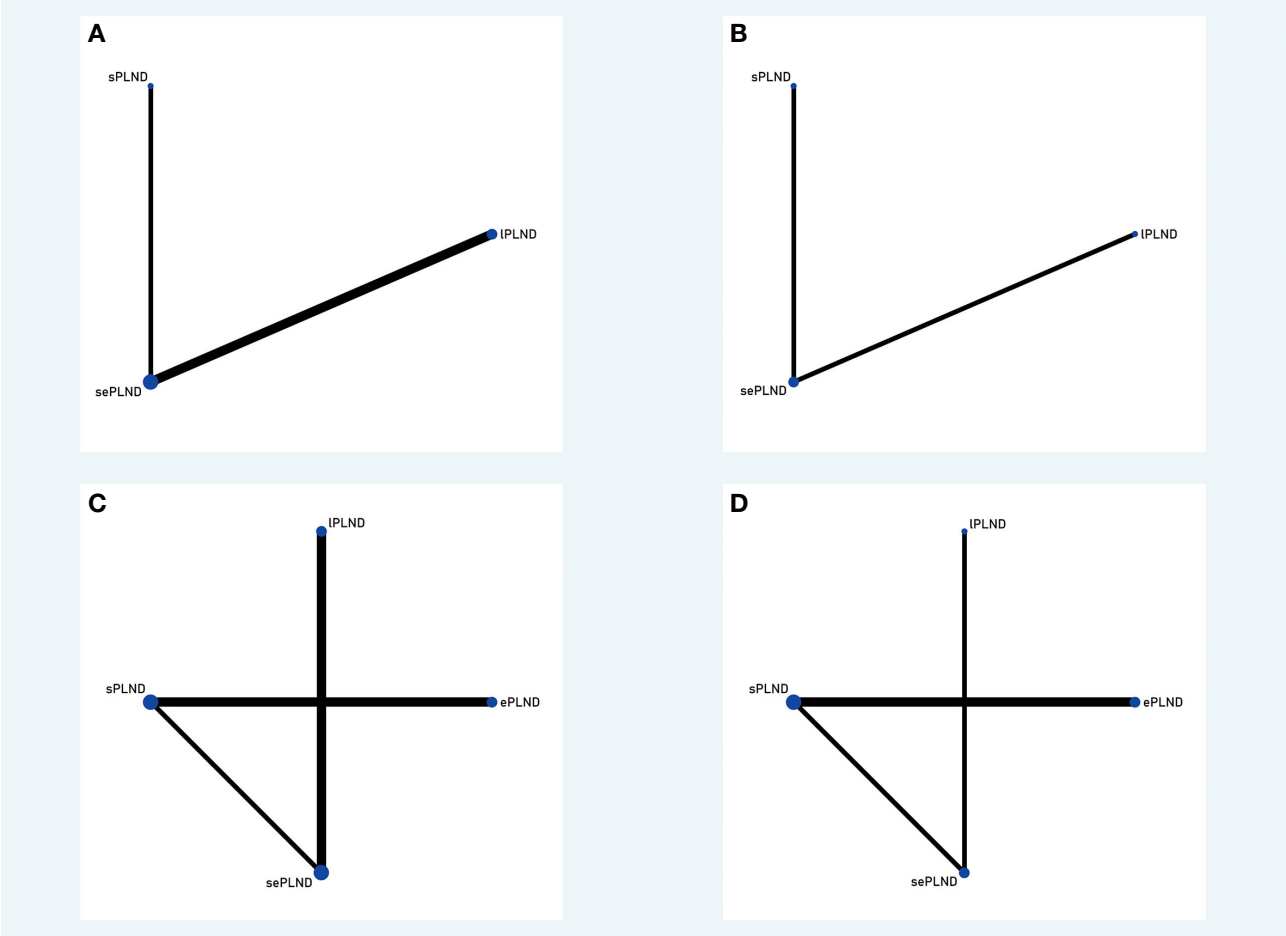
**(A)** Network plot showing the association of different PLND templates with the early Clavien-Dindo grade 5 complications rates in patients undergoing radical cystectomy. **(B)** Network plot showing the association of different PLND templates with the late Clavien-Dindo grade 5 complications rates in patients undergoing radical cystectomy. **(C)** Network plot showing the association of different PLND templates with the early Clavien-Dindo grade 3-4 complications rates in patients undergoing radical cystectomy. **(D)** Network plot showing the association of different PLND templates with the late Clavien-Dindo grade 3-4 complications rates in patients undergoing radical cystectomy. PLND, Pelvic lymph node dissection. lPLND, limited pelvic lymph node dissection. sPLND, standard pelvic lymph node dissection. ePLND, extended pelvic lymph node dissection. sePLND, super-extended pelvic lymph node dissection. Note: Lymph node dissection templates are represented by nodes and direct comparison trials between different templates are linked with a line. The area of the dot represents the sample size of each template and the width of the line corresponds the number of direct comparison trials.

We established a network meta-analysis to compare four different PLND templates. Comparisons among four different PLND templates in RFS, DSS and OS are summarized in **Figure 4**, and comparisons among four different PLND templates in major complications are summarized in **Figure 5**.

#### 3.5.1 RFS

**Figure 6:**
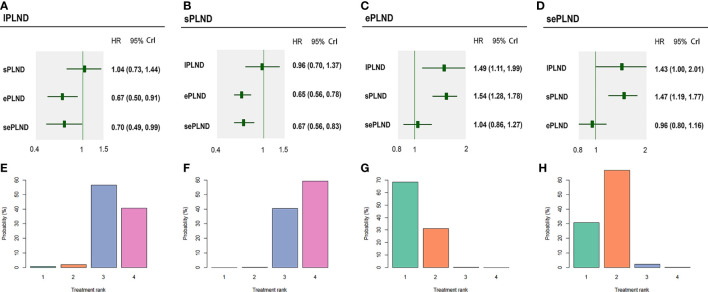
Bayesian network analysis results for RFS and rank probabilities of each PLND template based on the random effects model. **(A)** Other three PLND templates vs. lPLND template. **(B)** Other three PLND templates vs. sPLND template. **(C)** Other three PLND templates vs. ePLND template. **(D)** Other three PLND templates vs. sePLND template. **(E)** Probabilities of ranking the lPLND template in the first, second, third and fourth place among four PLND templates. **(F)** Probabilities of ranking the sPLND template in the first, second, third and fourth place among four PLND templates. **(G)** Probabilities of ranking the ePLND template in the first, second, third and fourth place among four PLND templates. **(H)** Probabilities of ranking the sePLND template in the first, second, third and fourth place among four PLND templates. RFS, Recurrence free survival. PLND, Pelvic lymph node dissection. HR, Hazard ratio. CrI, credible interval. lPLND, limited pelvic lymph node dissection. sPLND, standard pelvic lymph node dissection. ePLND, extended pelvic lymph node dissection. sePLND, super-extended pelvic lymph node dissection.

According to the results, ePLND and sePLND were statistically superior to lPLND in terms of RFS (HR: 0.67, 95% CrI: 0.50 to 0.91) (HR: 0.70, 95% CrI: 0.49 to 0.99). And we also found that ePLND and sePLND were statistically superior to sPLND in RFS (HR: 0.65, 95% CrI: 0.56 to 0.78) (HR: 0.67, 95% CrI: 0.56 to 0.83). But there was no significant difference between the lPLND group and the sPLND in RFS (HR: 1.04, 95% CrI: 0.73 to 1.44). And we did not find there was significant difference in RFS between the ePLND group and the sePLND group (HR: 1.04, 95% CrI: 0.86 to 1.27). Ranking on RFS indicated that ePLND had the highest probabilities (68.5%) to be the best option, followed by sePLND (30.7%), sPLND (0.7%) and lPLND (0.6%) (**Figure 6**).

#### 3.5.2 DSS

**Figure 7:**
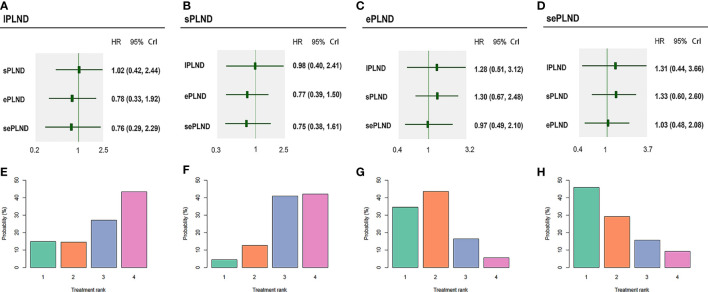
Bayesian network analysis results for DSS and rank probabilities of each PLND template based on the random effects model. **(A)** Other three PLND templates vs. lPLND template. **(B)** Other three PLND templates vs. sPLND template. **(C)** Other three PLND templates vs. ePLND template. **(D)** Other three PLND templates vs. sePLND template. **(E)** Probabilities of ranking the lPLND template in the first, second, third and fourth place among four PLND templates. **(F)** Probabilities of ranking the sPLND template in the first, second, third and fourth place among four PLND templates. **(G)** Probabilities of ranking the ePLND template in the first, second, third and fourth place among four PLND templates. **(H)** Probabilities of ranking the sePLND template in the first, second, third and fourth place among four PLND templates. DSS, Disease specific survival. PLND, Pelvic lymph node dissection. HR, Hazard ratio. CrI, credible interval. lPLND, limited pelvic lymph node dissection. sPLND, standard pelvic lymph node dissection. ePLND, extended pelvic lymph node dissection. sePLND, super-extended pelvic lymph node dissection.

We did not find any significant difference in DSS between any two PLND templates. Ranking on DSS showed that sePLND had the highest probabilities (45.9%) to be the best option, followed by ePLND (34.5%), lPLND (14.8%) and sPLND (4.4%) (**Figure 7**).

#### 3.5.3 OS

**Figure 8:**
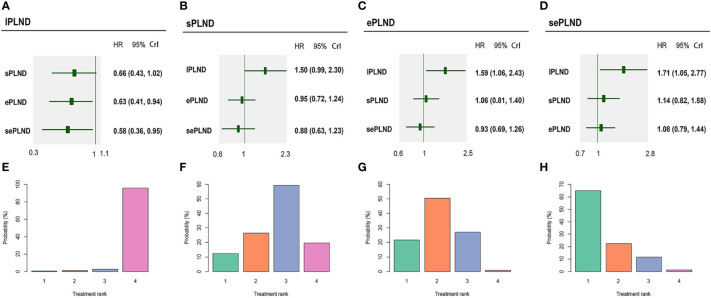
Bayesian network analysis results for OS and rank probabilities of each PLND template based on the random-effects model. **(A)** Other three PLND templates vs. lPLND template. **(B)** Other three PLND templates vs. sPLND template. **(C)** Other three PLND templates vs. ePLND template. **(D)** Other three PLND templates vs. sePLND template. **(E)** Probabilities of ranking the lPLND template in the first, second, third and fourth place among four PLND templates. **(F)** Probabilities of ranking the sPLND template in the first, second, third and fourth place among four PLND templates. **(G)** Probabilities of ranking the ePLND template in the first, second, third and fourth place among four PLND templates. **(H)** Probabilities of ranking the sePLND template in the first, second, third and fourth place among four PLND templates. OS, Overall survival. PLND, Pelvic lymph node dissection. HR, Hazard ratio. CrI, credible interval. lPLND, limited pelvic lymph node dissection. sPLND, standard pelvic lymph node dissection. ePLND, extended pelvic lymph node dissection. sePLND, super-extended pelvic lymph node dissection.

According to the results, ePLND and sePLND were statistically superior to lPLND in terms of OS (HR: 0.63, 95% CrI: 0.41 to 0.94) (HR: 0.58, 95% CrI: 0.36 to 0.95). But there was no significant difference between the sPLND group and the lPLND group in OS (HR: 0.66, 95% CrI: 0.43 to 1.02). And there was no significant difference between the sePLND group and ePLND group (HR: 0.93, 95% CrI: 0.69 to 1.26), the sePLND group and the sPLND group (HR: 0.88, 95% CrI: 0.63 to 1.23), the ePLND group and the sPLND group (HR: 0.95, 95% CrI: 0.72 to 1.24) in OS. Ranking on OS indicated that sePLND had the highest probabilities (65.1%) to be the best option, followed by ePLND (21.6%), sPLND (12.3%) and lPLND (0.6%). (**Figure 8**)

#### 3.5.4 Complications

**Figure 9:**
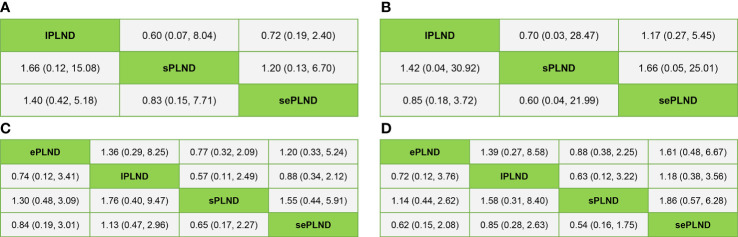
**(A)** Bayesian network analysis results for early Clavien-Dindo grade 5 complications rates of each PLND template based on the random effects model. **(B)** Bayesian network analysis results for late Clavien-Dindo grade 5 complications rates of each PLND template based on the random effects model. **(C)** Bayesian network analysis results for early Clavien-Dindo grade 3-4 complications rates of each PLND template based on the random effects model. **(D)** Bayesian network analysis results for late Clavien-Dindo grade 3-4 complications rates of each PLND template based on the random effects model. lPLND, limited pelvic lymph node dissection. sPLND, standard pelvic lymph node dissection. ePLND, extended pelvic lymph node dissection. sePLND, super-extended pelvic lymph node dissection.

**Figure 10:**
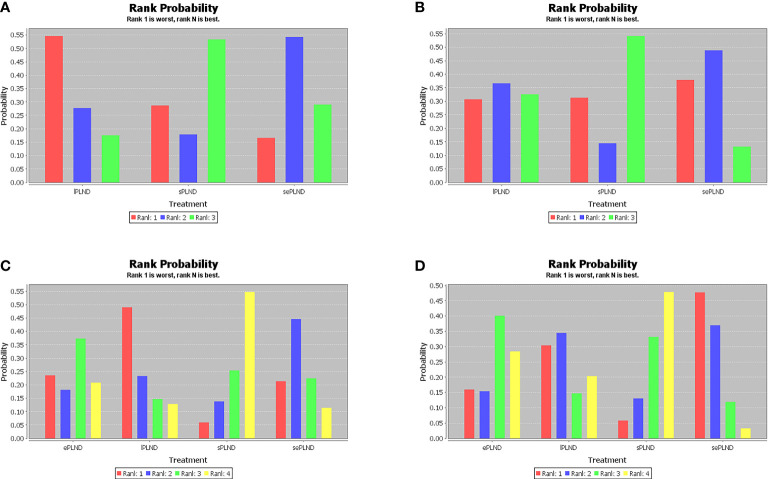
**(A)** Rank probabilities of each PLND template in the first, second, third and fourth place among three PLND templates for early Clavien-Dindo grade 5 complications rates. **(B)** Rank probabilities of each PLND template in the first, second, third and fourth place among three PLND templates for late Clavien-Dindo grade 5 complications rates. **(C)** Rank probabilities of each PLND template in the first, second, third and fourth place among four PLND templates for early Clavien-Dindo grade 3-4 complications rates. **(D)** Rank probabilities of each PLND template in the first, second, third and fourth place among four PLND templates for late Clavien-Dindo grade 3-4 complications rates. lPLND, limited pelvic lymph node dissection. sPLND, standard pelvic lymph node dissection. ePLND, extended pelvic lymph node dissection. sePLND, super-extended pelvic lymph node dissection.

When the grade 5 complications rate is 0, the study cannot be included in network meta-analysis using ADDIS (Aggregate Data Drug Information System) software, so some studies with the ePLND template have to be excluded from the indirect comparisons of early and late grade 5 complications rates. Based on the pooled results of other studies, we found that there was no significant difference in early grade 5 complications rates and late grade 5 complications rates between any two PLND templates in lPLND, sPLND and sePLND templates. (**Figure 9A, B**) Ranking on early grade 5 complications rates showed that sPLND had the highest probabilities (49%) to be the best option, followed by sePLND (30%) and lPLND (21%). (**Figure 10A**) Ranking on late grade 5 complications rates showed that sPLND had the highest probabilities (56%) to be the best option, followed by lPLND (29%) and sePLND (15%). (**Figure 10B**) And there was no significant difference in early grade 3-4 complications rates and late grade 3-4 complications rates between any two PLND templates. (**Figure 9C, D**) Ranking on early grade 3-4 complications rates showed that sPLND had the highest probabilities (55%) to be the best option, followed by ePLND (22%), sePLND (13%) and lPLND (11%). (**Figure 10C**) And ranking on late grade 3-4 complications rates showed that sPLND had the highest probabilities (51%) to be the best option, followed by ePLND (28%), lPLND (18%) and sePLND (3%). (**Figure 10D**) According to the results of node splitting analyses, there was no obvious inconsistency between direct comparisons and indirect comparisons.

### 3.6 Results of qualitative analysis

A study showed that the operation time of the sePLND group was significantly longer than that of the lPLND group (330 [225–410] min vs. 277 [205–300] min, p <0.01) (24). And another study showed that blood loss was higher in the sePLND group than that in the sPLND and in the ePLND groups (1000 [700-1200] ml vs. 700 [500-1225] ml vs.700 [400-1200] ml, p =0.08) (27). It indicated that sePLND involved longer operation time, higher risk, greater harm to patients, which made us negative about sePLND being the optimal template.

### 3.7 Inconsistency analysis

We conducted inconsistency analysis by comparing the difference in HRs between the direct comparisons and the indirect comparisons, and we found that all differences were less than 25% (**Table 2**), which showed that the results of our network meta-analyses were reliable.

**Table 2:** Inconsistency between direct evidence and indirect evidence.

Comparisons	HR for direct evidence	HR for indirect evidence	Inconsistency (%)	Level of inconsistency
RFS				
ePLND vs lPLND	0.67 (0.54, 0.82)	0.67 (0.50, 0.91)	0	Low
ePLND vs sPLND	0.64 (0.54, 0.76)	0.65 (0.56, 0.78)	1.56	Low
sePLND vs sPLND	0.73 (0.59, 0.90)	0.67 (0.56, 0.83)	-8.22	Low
sePLND vs ePLND	1.00 (0.85, 1.17)	1.04 (0.86, 1.27)	4.00	Low
DSS				
sPLND vs lPLND	0.99 (0.63, 1.55)	1.02 (0.42, 2.44)	3.03	Low
ePLND vs lPLND	0.81 (0.44, 1.48)	0.78 (0.33, 1.92)	-3.70	Low
ePLND vs sPLND	0.72 (0.41, 1.26)	0.77 (0.39, 1.50)	6.94	Low
sePLND vs sPLND	0.90 (0.50, 1.61)	0.75 (0.38, 1.61)	-16.67	Low
sePLND vs ePLND	0.78 (0.56, 1.09)	0.97 (0.49, 2.10)	24.36	Low
OS				
sPLND vs lPLND	0.62 (0.43, 0.91)	0.66 (0.43, 1.02)	6.45	Low
ePLND vs lPLND	0.65 (0.53, 0.81)	0.63 (0.41, 0.94)	-3.08	Low
ePLND vs sPLND	0.94 (0.73, 1.23)	0.95 (0.72, 1.24)	1.06	Low
sePLND vs sPLND	0.99 (0.58, 1.71)	0.88 (0.63, 1.23)	-11.11	Low
sePLND vs ePLND	0.85 (0.54, 1.34)	0.93 (0.69, 1.26)	9.41	Low

HR, Hazard Ratio.

## 4 Discussion

Survival outcomes and postoperative complications of patients undergoing RC with four different PLND templates were discussed in our review. Seventeen studies and 6503 patients were included in our quantitative analysis. The results of meta-analyses and Bayesian analyses showed that ePLND and sePLND were statistically superior to sPLND and lPLND in the RFS of patients, but not in DSS and OS. We found that there was no significant difference in complications rates between any two templates based on meta-analyses and Bayesian meta-analyses. And the treatment effect ranking on RFS, DSS and OS indicated that ePLND and sePLND had the first or second highest probabilities to be the best option. The complications rates ranking on early and late complications rates indicated that ePLND might be the better option than sePLND. Considering that sePLND involved longer operation time, higher risk, greater harm to patients (14), and sePLND may not result in better prognosis, we think that the ePLND template which dissect lymph nodes to aortic bifurcation area is sufficient for patients undergoing RC.

The optimal extent of lymph node dissection for RC has been controversial for a long time. There were several meta-analyses investigating which template is the best before, but due to the limited number of studies included, the reviewers regarded the lPLND template or the sPLND template as non-extended PLND main group, and regarded the ePLND template or the sePLND template as extended PLND main group. Bi et al. included 6 studies in their studies (35). And the results of the study showed that the extended PLND main group was associated with better RFS whatever the lymph node status. But for the patients who were in pT2 stage, there was no significant difference in RFS between the extended and the non-extended PLND main group. Another meta-analysis conducted by Mandel et al. (36) focused on 5-year recurrence-free survival and reached similar results. Wang et al. found the extended PLND main group had better RFS and DSS than the non-extended PLND main group, while there was no significant difference in OS, postoperative mortality and major postoperative complications between the two groups (37). At present, the only RCT that compared the lPLND template and the sePLND template was conducted by Gashend et al. in 2019 (29). And they found that the RFS, DSS and OS of the sePLND group were not significantly better than that of the lPLND group. So, we think that more RCTs and high-quality meta-analyses are needed to validate their findings.

We believe that there are still differences even between PLND with similar extents, and it is unreasonable to regard them as one single PLND template, especially when it comes to the superiority of ePLND and sePLND, about which there is still a lot of controversy. So, we separated four PLND templates by extents and discussed them separately in network meta-analyses using a Bayesian model. A Bayesian network meta-analysis can compare four templates at the same time, which is different from ordinary meta-analysis. In addition, even if there is no significant statistical difference between different templates, a network meta-analysis can still give ranking possibilities among different PLND templates, which may be important for making clinical decisions. We believe our study will help clinicians choose a specific, well-defined PLND template instead of vague extended PLND or non-extended PLND. The optimal PLND template we found should be the most beneficial to the patients under the consideration of all aspects. In addition, our study also quantitatively analyzed the survival outcomes of ePLND and sePLND group, which were not covered in the previous studies. With the method of Tierney et al., we extracted data from some studies which only presented the survival curves and not included in previous studies (19). We also included some newly published articles, which would further improve the credibility of our conclusions. The meta-analyses mentioned before (35–37) chose the analysis models based on the heterogeneity of the pooled results. If the heterogeneity was less than 50%, the fixed effects model was employed for analysis, otherwise the random effects model was used. In our study, in order to reach a more conservative and practical conclusion to guide clinical practice, we used the random effects model for Bayesian analysis.

In 30% to 40% of BCa patients, lymph nodes are the only site of metastases (38). Extended PLND can reduce the risk of recurrence by eliminating micro-metastases in lymph nodes to improve the RFS. These micro-metastases residing in lymph nodes are sometimes difficult to distinguish both clinically and microscopically (39). In this way, ePLND and sePLND can improve the RFS in patients whose lymph node status are cN0. The presence of lymph node metastases above the bifurcation of the common iliac artery have been reported in about 40% of patients with lymph node metastases, but lymph node metastases above the aortic bifurcation are rare (40, 41). So, patients undergoing sePLND that extends to the level of the inferior mesenteric artery did not get better RFS than those undergoing ePLND. Normally, a benefit in DSS should be consistent with a benefit in RFS, this is different from our results, which may be due to the small number of studies we included and the quality of the articles we included are not very high. It is one of the main shortcomings of our study. In general, OS are affected by more factors than RFS and DSS; like age, gender, pT stage and variant histology (42), and the influence of adjuvant and neoadjuvant therapy is important, too. So, we found there was no significant difference in OS among some patients undergoing different PLND templates.

Except the extent of lymph node dissection, some studies have shown that the dissected lymph nodes number and lymph node ratio (LNR) may also have an impact on prognosis (43–45). A sufficient number of lymph nodes helped to provide more accurate information of lymph node status, which could guide clinical practice. More lymph nodes can be obtained by performing a more extensive PLND template. However, the cut-off value for the minimum number of lymph nodes has not been determined yet (46, 47). LNR is the proportion of positive lymph nodes in all lymph nodes (48). Poorer prognosis may be associated with higher LNR. Even though some studies have shown that LNR is associated with the survival outcomes of patients (44), LNR is no longer considered to be an accurate prognostic factor according to EAU guidelines (2021), so we are skeptical about the value of LNR in predicting the prognosis of patients.

Based on the results of the inconsistency analysis, we found the inconsistency between the direct and indirect evidence in our study was small. However, it is worth noting that the direct evidence showed that the sPLND group was associated with better OS compared to the lPLND group (0.62, 95% CI: 0.43 to 0.91), while the indirect result showed that there was just no significant difference between the two groups (0.66, 95% CrI: 0.43 to 1.02). The reason for it might be that only one study assessed the difference in OS between the sPLND group and the lPLND group which may lead some bias, and the difference may be underestimated because a more conservative random effects model was used in Bayesian analysis. In general, the inconsistency of our study was small, our quantitative analyses were stable.

Although our study yielded some promising results, there were also some limitations in this review. First, most studies we included were cohort studies, physicians’ choices of PLND could be influenced by many factors. We need more high-quality multicenter RCTs with large sample sizes to validate our conclusions. Second, when direct comparisons are made, we found high heterogeneity when pooling estimates for some results. Heterogeneity may be due to differences in pathological staging and adjuvant therapy among patients. Besides, we did not conduct subgroup analyses in the direct comparisons since the number of studies comparing any two templates is relatively small, and there are few articles report the situation of clinically visible or suspicious lymph node metastases. Subgroup analysis may explain the source of heterogeneity and help us identify the patients who need ePLND or even PLND more. Therefore, future studies should include more homogeneous patients to confirm the existing conclusions. Third, for some studies did not provide HRs and 95% CIs, we used the method of Tierney et al. to estimate HRs and 95% CI, which might introduce some bias. Despite these insufficiencies, we came to different conclusions from previous studies, which have implications for determining the optimal extent of lymph node dissection for radical cystectomy.

## 5 Conclusion

Patients undergoing sePLND and ePLND templates had better RFS, but no better DSS or OS, than those undergoing lPLND or sPLND templates, however, survival outcomes did not differ between patients undergoing sePLND or ePLND templates. Considering that sePLND involves longer operation time, higher risk, and greater degree of difficulty than ePLND, and performing sePLND may not result in better prognosis, so it seems that there is no need for seLPND. We think that ePLND might be the optimal lymph node dissection template for radical cystectomy.

## Data availability statement

The data that supports the findings of this study are available from the corresponding author upon reasonable request.

## Author contributions

(I) Conception and design, WQ and SC; (II) Administrative support, SC and BS; (III) Provision of study materials or patients, WQ and MZ; (IV) Collection and assembly of data, WQ, MZ, NJ, and YZ; (V) Data analysis and interpretation, WQ, MZ, and NJ; (VI) Manuscript writing, all authors; (VII) Final approval of manuscript, all authors. All authors contributed to the article and approved the submitted version.

## Funding

National Natural Science Foundation of China (Grant No. 81900637 to BS, Grant No. 81800672 to SC), the Tai Shan Scholar Foundation (ts201511092 to BS), the Primary Research & Development Plan of Shandong Province (2019GSF108123 to SC) and the Beijing Bethune Charitable Foundation (mnzl202020 to SC).

## Conflict of interest

The authors declare that the research was conducted in the absence of any commercial or financial relationships that could be construed as a potential conflict of interest.

## Publisher’s note

All claims expressed in this article are solely those of the authors and do not necessarily represent those of their affiliated organizations, or those of the publisher, the editors and the reviewers. Any product that may be evaluated in this article, or claim that may be made by its manufacturer, is not guaranteed or endorsed by the publisher.
