# Supplementary material for: Which lymph node dissection template is optimal for radical cystectomy? A systematic review and Bayesian network meta-analysis
Source: Front Oncol. 2022 Nov 25;12:986150. doi: 10.3389/fonc.2022.986150 (PMC9732561; doi:10.3389/fonc.2022.986150)

Supplementary Material

# Supplementary material S1 Details of search strategy for all databases.

**PubMed：**

((Lymph Node Excision）OR (Excision, Lymph Node) OR (Excisions Lymph Node) OR (Lymph Node Excisions) OR (Lymphadenectomy) OR (Lymphadenectomies) OR (Lymph Node Dissection) OR (Dissection, Lymph Node) OR (Dissections, Lymph Node) OR (Lymph Node Dissections) OR (Node Dissection, Lymph ) OR (Node Dissections, Lymph)) AND ((Radical Cystectomy) OR (Radical Cystectomies))

**Embase:**

#1 'cystectomy'/exp

#2 'bladder extirpation' OR 'bladder resection' OR 'cystectomy, total' OR 'cystoprostatectomy' OR 'pericystectomy' OR 'radical cystectomy' OR 'total cystectomy' OR 'urinary bladder resection' 17,604

#3 #1 OR #2 34,572

#4 'lymph node dissection'/exp 88,077

#5 'lymph nodal dissection' OR 'lymph node excision' OR 'lymph node extirpation' OR 'lymph node resection' OR 'lymphadenectomy' OR 'lymphoadenectomy' 35,285

#6 #4 OR #5 93,307

#7 #3 AND #6 AND ([article]/lim OR [article in press]/lim OR [conference paper]/lim OR [data papers]/lim OR [short survey]/lim OR [preprint]/lim) AND [english]/lim 1790

**Cochrane**

#1 MeSH descriptor: [Lymph Node Excision] explode all trees 1398

#2 Lymphadenectomies OR Lymphadenectomy OR (Excision, Lymph Node) OR (Lymph Node Excisions) OR (Excisions, Lymph Node) OR (Lymph Node Dissection) OR (Dissection, Lymph Node) OR (Dissections, Lymph Node) OR (Node Dissection, Lymph) OR (Node Dissections, Lymph) OR (Lymph Node Dissections) 5227

#3 #1 OR #2 5430

#4 MeSH descriptor: [Cystectomy] explode all trees 285

#5 Cystectomies 40

#6 #4 OR #5 315

#7 #3 AND #6 32

**2 Supplementary material S2** The we used in OpenBUGS

model{

#Define Prior Distributions

#on random tx effect variance

sd~dunif(0,5)

reTau < - 2/pow(sd,2)

#On tx effect mean

beta[1] < -0

for (tt in 2:nTx){

beta[tt]~dnorm(0,1.0E-6)

}

#On individual study baseline effect

for(ss in 1:nStudies){

alpha[ss] ~ dnorm(0,1.0E-6)

}

#Define random effect

for (ss in 1:nStudies){

for(tt in 1:nTx){

re[ss,tt]~dnorm(0,reTau)

}

}

#Fit data

#For hazard ratio reporting studies

for(ii in 1:LnObs ){

Lmu[ii] < - alpha[Lstudy[ii]]*multi[ii] + re[Lstudy

[ii],Ltx[ii]] -

re[Lstudy[ii],Lbase[ii]] + beta[Ltx[ii]] - beta

[Lbase[ii]]

Lprec[ii] < - 1/pow(Lse[ii],2)

Lmean[ii] ~ dnorm(Lmu[ii],Lprec[ii])

}

# Calculate HRs

for (hh in 2:nTx) {

hr[hh] < -exp(beta[hh])

}

# Ranking plot

for (ll in 1:nTx) {

for (mm in 1:nTx) {

rk[ll,mm] < - equals(ranked(beta[],mm),beta[ll])

}

}

}

# Data

# Data set descriptors

list(LnObs = 13, nTx = 4, nStudies = 12)

# Log hazard ratio and log hazard data

Lstudy[] Ltx[] Lbase[] Lmean[] Lse[] multi[]

1 2 1 -0.20992 0.262398 0

2 3 2 0.41893 0.114241 0

…

12 4 1 -0.31883 0.578859 1

END

# Initial values

list(alpha = c(-0.50,-0.50,-0.50,-0.50,-0.50,-0.50,-0.50,-0.50,-0.50,-0.50,-0.50,-0.50), beta =

c(NA,-0.5,-0.5,-0.5), sd=0.1)

list(alpha = c(0.50,0.50,0.50,0.50,0.50,0.50,0.50,0.50,0.50,0.50,0.50,0.50), beta = c(NA,0.5,0.5,0.5), sd=1)

list(alpha = c(0,0,0,0,0,0,0,0,0,0,0,0), beta = c(NA,0,0,0), sd=1)

**3 Supplementary material S3** Detailed quality assessments of cohort studies

**S3.1 Detailed quality assessment of cohort study**

| Items of NOS | Studies | | | | | | | |
| --- | --- | --- | --- | --- | --- | --- | --- | --- |
|  | Poulsen | Brössner | Dhar | Holmer | Hugen | Abol-Enein | Dharaskar | Zehnder |
| Selection |  |  |  |  |  |  |  |  |
| Representativeness of the exposed cohort | * | * | * | * | * | * | * | * |
| Selection of the non-exposed cohort | * | * | * | * | * | * | * | * |
| Ascertainment of exposure | * | * | * | * | * | * | * | * |
| Demonstration that outcome of interest was not present at start of study |  |  | * |  | * | * | * | * |
| Comparability |  |  |  |  |  |  |  |  |
| Comparability of cohorts on basis of the design or analysis | ** | * | ** | ** | * | * | * | * |
| Outcome |  |  |  |  |  |  |  |  |
| Assessment of outcome | * | * | * | * | * | * | * | * |
| Was follow-up long enough for outcomes to occur |  | * |  |  | * | * |  | * |
| Adequacy of follow up of cohorts |  |  | * |  | * | * | * | * |
| Total | 6 | 6 | 8 | 6 | 8 | 8 | 7 | 8 |

A study can be awarded a maximum of one star for each numbered item within the Selection and Outcome categories. A maximum of two stars can be given for Comparability. Studies rates ≥6 are eligible. NOS, Newcastle-Ottawa Scale.

**S3.2**

| Items of NOS |  | Studies | | | | | | | | |
| --- | --- | --- | --- | --- | --- | --- | --- | --- | --- | --- |
|  | Zhu | Simone | Mata | Pedrosa^a^ | Abdi | Møller | Brunocilla | Choi | D’Andrea |  |
| Selection |  |  |  |  |  |  |  |  |  |  |
| Representativeness of the exposed cohort | * | * | * | * | * | * | * | * | * |  |
| Selection of the non-exposed cohort | * | * | * | * | * | * | * | * | * |  |
| Ascertainment of exposure | * | * | * |  | * | * | * | * | * |  |
| Demonstration that outcome of interest was not present at start of study | * | * | * |  |  | * | * | * |  |  |
| Comparability |  |  |  |  |  |  |  |  |  |  |
| Comparability of cohorts on basis of the design or analysis | * | * | * | * | ** | * | * | * | * |  |
| Outcome |  |  |  |  |  |  |  |  |  |  |
| Assessment of outcome | * | * | * |  | * | * | * | * | * |  |
| Was follow-up long enough for outcomes to occur |  | * |  | * |  |  | * |  | * |  |
| Adequacy of follow up of cohorts | * | * | * | * | * | * | * | * | * |  |
| Total | 7 | 8 | 7 | 5 | 7 | 7 | 8 | 7 | 7 |  |

A study can be awarded a maximum of one star for each numbered item within the Selection and Outcome categories. A maximum of two stars can be given for Comparability. Studies rates ≥6 are eligible. X^a^ not qualified study. NOS, Newcastle-Ottawa Scale.

# 4 Supplementary material S4 Quality assessment of the randomized controlled trial


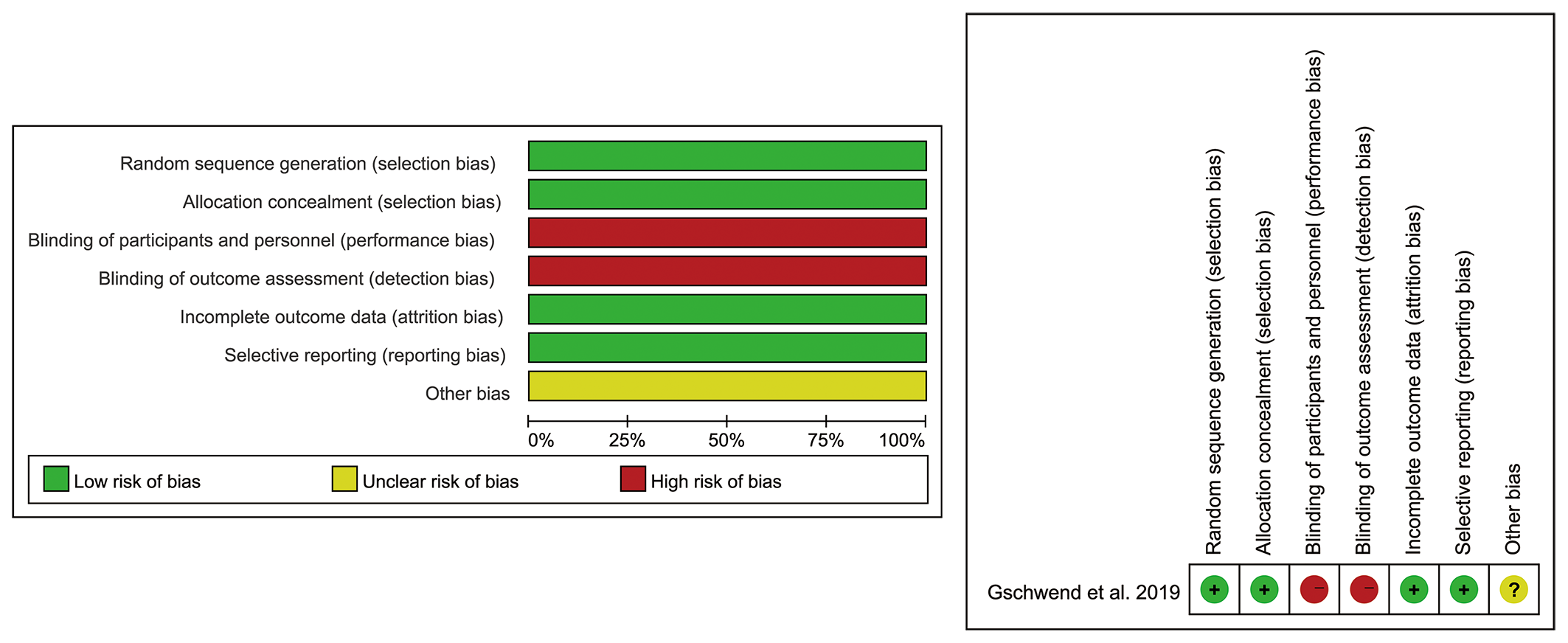

Supplement: Supplementary file 1 [file DataSheet_1.docx]
